# Influence of Osteopontin-Coated Surface on Osteoblast Behaviour Relevant to Dental Implant Integration

**DOI:** 10.1155/ijod/9929498

**Published:** 2025-05-23

**Authors:** Jira Chansaenroj, Nunthawan Nowwarote, Chenphop Sawangmake, Panida Thanyasrisung, Kornkanok Khlongwanitchakul, Chanya Srisa, Nattakarn Povichit, Prasit Pavasant, Thanaphum Osathanon

**Affiliations:** ^1^Center of Excellence for Dental Stem Cell Biology, Faculty of Dentistry, Chulalongkorn University, Bangkok, Thailand; ^2^INSERM UMR1163, Imagine Institute, Universite Paris Cite, Paris, France; ^3^Department of Oral Biology, Faculty of Dentistry, Universite Paris Cite, Paris, France; ^4^Department of Pharmacology, Faculty of Veterinary Science, Chulalongkorn University, Bangkok, Thailand; ^5^Department of Microbiology and Research Unit on Oral Microbiology and Immunology, Faculty of Dentistry, Chulalongkorn University, Bangkok, Thailand; ^6^Center of Excellence for Regenerative Dentistry and Department of Anatomy, Faculty of Dentistry, Chulalongkorn University, Bangkok, Thailand

**Keywords:** cell attachment, cell spreading, osteoblast, osteopontin, titanium

## Abstract

Osteopontin (OPN) plays an important role in osteogenesis mechanisms and influences bone formation and resorption. OPN contains several bioactive domains, including a hydroxyapatite (HA) binding domain and an osteoinductive sequence Ser–Val–Val–Tyr–Gly–Leu–Arg (SVVYGLR). The present study coated the OPN at concentrations of 0.5, 1.0 and 1.5 µg/mL on the glass surface to examine the effects of adhesion and spreading of a murine osteoblast cell line. After cells were seeded on the modified surfaces. Cell number and spreading were evaluated using the MTT assay and phalloidin immunocytochemistry staining. The cell attachment was examined after seeding for 1 h. Although a slight increase in cell attachment was noted in the 1.5 µg/mL OPN-coated condition. There was no statistically significant difference. Cell proliferation was significantly increased in 1 and 1.5 µg/mL OPN-coated conditions at day 7. Cell spreading was markedly noted in OPN-coated conditions compared with the control at 10 min and 3 h. However, cell morphology was similar among conditions at 24 h. Therefore, OPN could be useful for material surface modification to promote osteoblast responses.

## 1. Introduction

Osteopontin (OPN) is a highly glycosylated and phosphorylated sialoprotein and belongs to a small integrin-binding ligand N-linked glycoprotein (SIBLING) family [1–3]. It is one of the important extracellular matrices of mineralised tissues [2, 3]. OPN is a negatively charged protein, consisting of several domains. These domains exhibit different biological functions in the regulation of cell responses. OPN is encoded by the secreted phosphoprotein 1 (SPP1) gene on human chromosome 4q21 [1]. OPN interacts with integrin receptors via the Arg–Gly–Asp (RGD) motif [4]. OPN is highly expressed during the early healing phase of bone defects [5]. OPN null mice exhibit the impairment of collagen exposure to hydroxyapatite (HA), implying the crucial role of OPN in interfacial adhesion [6].

OPN contains crucial functional domains, including the HA binding domain and RGD-containing cell binding domain [7]. In addition, OPN also contains a binding sequence Ser–Val–Val–Tyr–Gly–Leu–Arg (SVVYGLR) located adjacent to the RGD-motif after thrombin cleavage [8]. SVVYGLR peptide promotes cell adhesion and proliferation of human mesenchymal stem cells in vitro, and SVVYGLR peptide-containing atelocollagen sponge enhances in vivo bone regeneration via osteoclastogenesis suppression in the rat calvarial defect model [9]. The osteointegrative effect of HA nanoparticles functionalised by OPN-containing poly-D, L-lactic-acid matrix is confirmed in the canine model as a significant increase in new bone formation in implant porosities [7]. Besides, the RGD peptide-coated surface of titanium intramedullary screws significantly enhances the number of OPN-positive osteoblasts residing around the implant [10]. Moreover, a newly formed lamellar bone around the RGD-coated titanium surface is significantly higher than the uncoated control at day 28 postimplantation, suggesting the acceleration of bone regeneration and remodelling [10]. These studies indicate the crucial effects of OPN on cell behaviours, especially osteoblasts. The present study aimed to examine the effects of an OPN-coated surface on cell adhesion, proliferation, and spreading of the murine preosteoblast cell line, MC3T3-E1.

## 2. Materials and Methods

### 2.1. OPN Expression and Purification

An expression plasmid pQE-30 containing histidine-tagged OPN was a generous gift from Professor Cecilia Giachelli, University of Washington, USA [11]. The plasmid was transformed into *Escherichia coli* JM109. Bacteria were cultured to the mid-log phase (OD_600 nm_ ≈ 0.5) using Luria–Bertani culture medium containing 100 µg/mL ampicillin. Protein expression and purification were performed in native conditions according to the manufacturer's protocol (Qiagen, USA). Briefly, isopropyl-*ß*-D-thiogalactoside (1 mM) was employed to induce histidine-tagged OPN expression. After 6 h of 37°C incubation, cells were harvested and resuspended in lysis buffer solution composed of 50 mM NaH_2_PO_4_, 300 mM NaCl and 10 mM imidazole at pH 8.0. The bacterial cells were disrupted using a sonicator until the suspension became clear. After centrifugation, the supernatant was mixed with Ni–NTA affinity chromatography (Qiagen, Valencia, CA, USA) at 4°C for 60 min. The column was washed three times with a wash buffer solution. The bound protein was eluted with an elution buffer solution. The active fractions were pooled and dialysed against 0.1 M phosphate buffer (pH 6.8). Purified OPN was lyophilised and stored at −80°C.

### 2.2. OPN-Coated Surface Preparation

Cover glasses were employed as the substrate surfaces for OPN coating in 24-well plates. The lyophilised OPN at concentrations of 0.5, 1.0 and 1.5 μg/mL was dissolved in sterile phosphate-buffered saline (PBS) and incubated with covered glass slides for 18 h at room temperature.

### 2.3. Enzyme-Linked Immunosorbent Assay (ELISA)

Specimens were washed with PBS and incubated with 10% horse serum for 1 h at room temperature to prevent nonspecific binding. Subsequently, specimens were incubated with primary antibody (rabbit antihuman OPN; Chemicon International, USA) for 2 h. The secondary antibody (goat antirabbit IgG; Abcam, USA) was incubated with the surfaces for 2 h. Further, the surfaces were exposed to streptavidin-HRP (cell signalling company, USA) for 1 h. ELISA washing buffer was used to rinse the surfaces between each step. ELISA substrate was added, and the reaction was stopped using a stop reagent. The absorbance was measured using a microplate reader at a wavelength of 450 nm.

### 2.4. Cell Culture

MC3T3-E1 cells were cultured in alpha MEM (Hyclone, USA) supplemented with 2 mM glutamine (Gibco, USA), 100 unit/mL penicillin (Gibco, USA), 100 µg/mL streptomycin (Gibco, USA), 0.25 µg amphotericin B (Gibco, USA), and 10% fetal bovine serum (FBS; Gibco, USA). Cells were incubated at 37°C in a humidified atmosphere containing 5% CO_2_. The condition medium was changed every 3 days. When the cells reached confluence, cells were trypsinized and subcultured at a 1:3 ratio.

### 2.5. Cell Viability Assay

Cells (10,000 cells) were seeded on an OPN-coated surface, and cell viability was evaluated using an MTT assay on days 1, 3 and 7. MTT solution was added to the culture medium without phenol red and incubated for 15 min, allowing the formation of formazan crystals. The precipitated formazan crystals were solubilised in glycine buffer solution and dimethylsulphoxide (prepared at a 1:9 ratio). The absorbance was further examined using a microplate reader at 570 nm.

### 2.6. Immunofluorescence Staining

Cells were fixed in 3% glutaraldehyde (Fluka Analytical, USA) for 10 min and washed twice with PBS. The phalloidin antibody (Invitrogen, USA) was prepared in 10% horse serum at a 1:100 dilution and then incubated with the samples for 15 min at room temperature. The fluorescence was evaluated by a fluorescence microscope (Zeiss, Germany) at 10 min, 3 h and 24 h.

### 2.7. Statistical Analyses

Results were shown as the mean ± standard error of the mean (SEM). The graphical illustration and statistical analyses were performed using Prism 8 (GraphPad Software, CA, USA). ANOVA followed by a Tukey's post hoc test was employed. The statistically significant difference was considered if the *p* < 0.05.

## 3. Results

### 3.1. Characterisation of the OPN-Coated Surface

**Figure 1:**
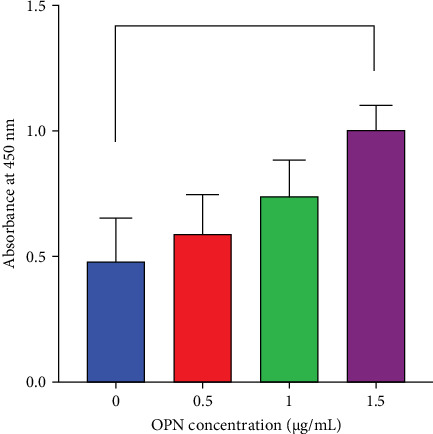
The amount of Osteopontin (OPN) absorbed on the glass surface at various concentrations. OPN was coated on glass surfaces at 0.5, 1.0 and 1.5 μg/mL concentrations. The uncoated surface was used as the control. Enzyme-linked immunosorbent assay was performed to detect bound OPN on the surfaces. The graph demonstrated the absorbance at 450 nm. The asterisk indicated a statistical significance (*p* < 0.05).

After coating OPN on a glass surface, an ELISA assay was performed to confirm the presence of bound OPN protein. Results showed that the amount of absorbed OPN on the glass surface increased dose-dependently (Figure 1). A significant amount of OPN on the glass surface was observed when coated on glass substrates with 1.5 µg/mL of OPN solution.

### 3.2. OPN Influences Cell Attachment and Proliferation

**Figure 2:**
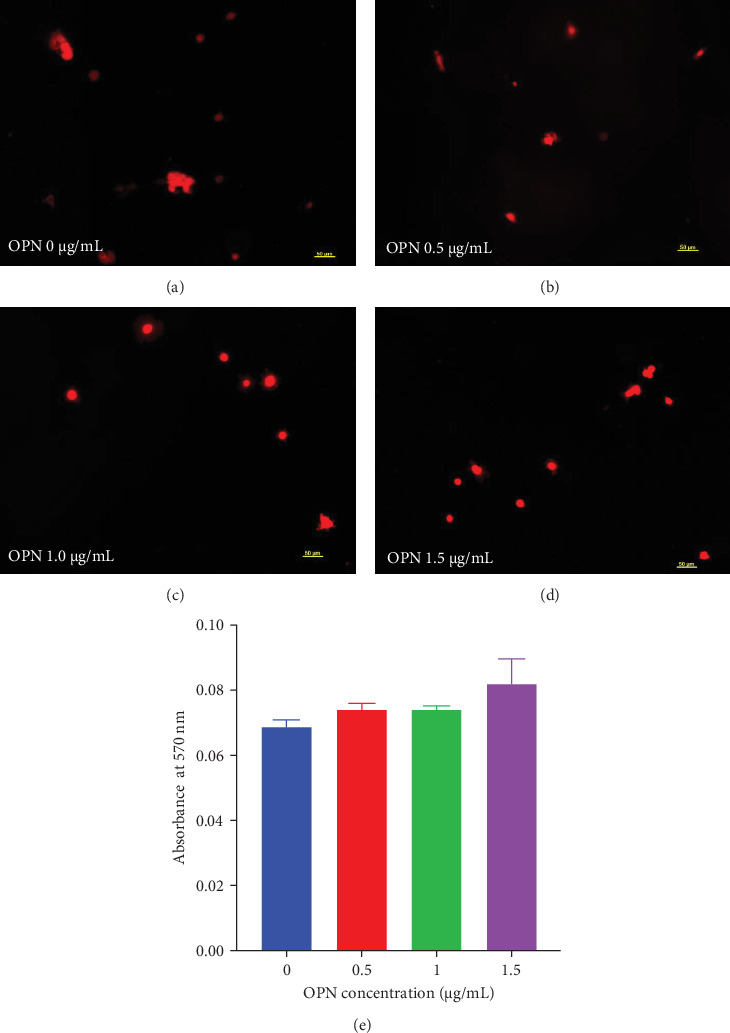
Cell attachment evaluated on OPN coated surfaces at various concentrations. OPN was coated on glass surfaces at 0.5, 1.0 and 1.5 μg/mL concentrations. The uncoated surface was used as the control. At 1 h, cells were fixed and stained with phalloidin. Representative images of attached cells were shown (A–D). MTT assay was performed (E).

Cell attachment was evaluated using image analysis and MTT assay. Cells were seeded on OPN-coated surfaces for 1 h and were subsequently stained with phalloidin (Figure 2A–D). There was no marked difference in cell attachment among conditions. MTT assay was also performed to confirm cell viability quantitatively. At 1 h after seeding cells on the OPN-coated surface, there was no significant difference in cell viability among groups (Figure 2E), although a slight increase in cells was observed in the OPN-coated groups.

**Figure 3:**
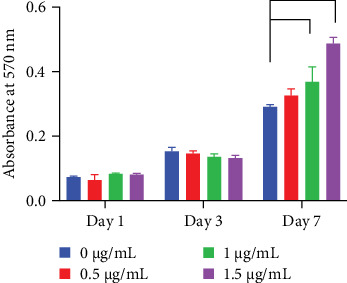
Cell viability assay on various concentrations of OPN coated surfaces. OPN was coated on glass surfaces at 0.5, 1.0 and 1.5 μg/mL concentrations. The uncoated surface was used as the control. MTT assay was performed, and the absorbance at 570 nm was measured on days 1, 3 and 7. The asterisk indicated a statistical significance (*p* < 0.05).

For the cell proliferation assay, cells were seeded on OPN-coated surfaces for 1, 3 and 7 days. Cell proliferation assay demonstrated that OPN at a concentration of 1 and 1.5 µg/mL significantly enhanced cell proliferation at day 7 compared with the control (Figure 3).

### 3.3. Effects of OPN on Cell Spreading

**Figure 4:**
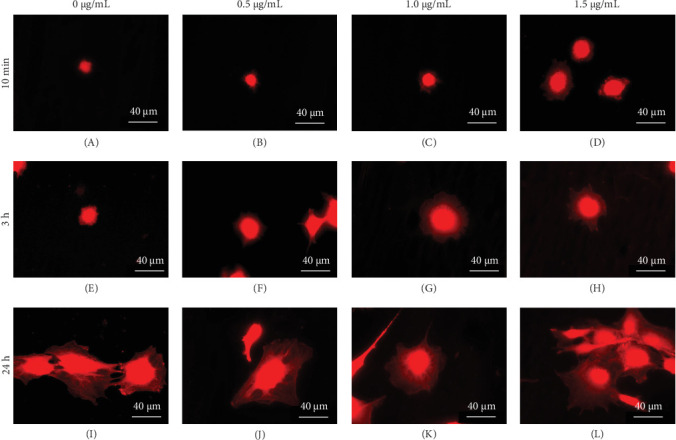
Spreading images of cell seed on various concentrations of OPN coated surfaces. OPN was coated on glass surfaces at 0.5, 1.0 and 1.5 μg/mL concentrations. The uncoated surface was used as the control. At 10 min (A–D), 3 h (E–H) and 24 h (I–L), cells were stained with phalloidin and examined using a fluorescence microscope. Representative images illustrate cell spreading morphology on different surfaces.

Cells were seeded on OPN-coated surfaces for 10 min, 3 h and 24 h. Subsequently, cells were stained with phalloidin. High-magnification images were randomly captured from various surface groups. The representatives of cell spreading images were demonstrated. Results demonstrated that cells in OPN-coated conditions exhibited better cell spreading than those in the control at 10 min and 3 h (Figure 4A–H). However, there was no dramatic difference at 24 h (Figure 4I–L).

## 4. Discussion

The present study described the influence of OPN-coated surfaces on murine preosteoblast behaviours, focusing on cell attachment and spreading. By incubating the OPN protein solution on the glass surface, OPN was absorbed on the surface, as confirmed by an ELISA assay. The result showed that the concentration of absorbed OPN was increased, corresponding to the OPN concentration used for coating, implying the success of the OPN coating procedure. Although surface characterisation techniques such as scanning electron microscopy, atomic force microscopy (AFM), and contact angle measurements were not employed in this study to assess the uniformity or thickness of the OPN coating. However, the concentration of OPN adsorbed onto the material surface was robustly quantified using an ELISA assay. ELISA provided sensitive and specific quantitative data on the amount of protein present, offering strong evidence of coating efficiency. In addition, the indirect observation of the consistent cell responses to the different OPN-coated concentrations implies the coating efficacy. However, as ELISA does not provide spatial distribution or morphological details, future investigations should incorporate surface characterisation techniques to validate coating uniformity and structural properties.

OPN contains RGD sequences, which could promote cell attachment [12]. However, the significant difference in cell attachment between the OPN-coated surface and the control surface was not noted in the present study, as evaluated at 1 h. This may be due to several reasons. First, the concentration of OPN utilised in the present study was relatively low compared to other studies [12, 13]. Thus, the low available domains are introduced to cells, which may result in poor cell attachment properties. Second, it has been reported that the OPN orientation and conformation influenced MC3T3 attachment behaviours [13]. In this regard, the regulation of OPN orientation/conformation via binding to the collagen resulted in higher MC3T3-E1 attachment than normal OPN-absorbed surfaces [13]. Third, the substratum chemistry also influenced the function of OPN [14, 15]. It has been illustrated that OPN coated on amine and carboxylic surface promoted better cell attachment than methyl surface [15]. In the present study, we employed a simple absorption method to bind OPN to the glass surface. Therefore, the orientation and conformation of the absorbed OPN were not controlled. Further study regarding the determination of orientation and conformation of absorbed OPN on surfaces should be performed.

Cell viability was evaluated using an MTT assay to indirectly infer cell viability. MTT assay aims to determine cell metabolic activity, and this indirectly implicates cell proliferation in vitro [16]. The present study demonstrated that OPN supported MC3T3-E1 proliferation at a concentration of 1.5 μg/mL at day 7, while there was no significant difference in early time points. Corresponding to the previous study, MG63 cells exhibited a significant increase in cell proliferation on an OPN (10 μg/mL) coated tissue culture plate compared with the control [12]. In addition, OPN overexpression in human embryonic kidney-293 cells increased cell proliferation as determined by BrdU incorporation and cell cycle analysis [17]. It has been shown that the influence of OPN on cell proliferation may depend on the presence of other growth factors. For example, OPN promoted human prostate cancer cells only when epidermal growth factor was also presented to the cells [18]. Together, OPN may regulate cell proliferation. However, the intracellular mechanism needs further investigation.

OPN promotes cell spreading. In this regard, previous works demonstrated that OPN facilitates the spreading of human aortic medial smooth muscle cells. In this respect, human aortic medial smooth muscle cells spread at 60 min on the OPN surface, but it takes 90 min for these cells to spread on the control surface [19]. In addition, the focal adhesion formation was observed on the OPN surface [19]. Further, these OPN-coated HAs enhanced osteoblast-like cell attachment and spreading [20]. In this respect, cells on the OPN-coated HA exhibit flattened morphology. Further, the average cell area on rhOPN-coated HA was approximately 4 times higher than on uncoated HA [20]. Correspondingly, the present study showed that cells seeded on an OPN-coated surface (1.5 μg/mL) exhibited better cell spreading compared with those cells on the control glass surface at 1 h.

Cell spreading could further influence cell differentiation [21, 22]. In this regard, RGD anchoring titanium surfaces improved cell adhesion and the spreading of osteoblast-like cells [23]. Correspondingly, these RGD anchoring surfaces enhanced an in vitro mineralisation compared with those noncoated surfaces [23]. The investigation of coated OPN influencing preosteoblast differentiation should be further performed.

To address current limitations and enhance the functional outcomes of OPN-coated surfaces, future investigations should incorporate surface characterisation techniques to further validate the uniformity and structural properties of the coating on specific materials that demonstrate high potential for use in in vivo studies and optimise the coating parameters, including techniques such as oriented immobilisation strategies using collagen-binding domains or surface chemistries tailored. Promoting bioactive OPN presentation may significantly improve cell adhesion and signalling outcomes. Combining OPN with other osteoinductive cues or growth factors could potentiate its effect on osteoblast proliferation and differentiation. Moreover, the advanced surface characterisation methods may help elucidate the bio-interface, and aid in designing more effective biomimetic coatings and exploration of downstream signalling pathways and transcriptional changes associated with OPN-mediated effects could offer deeper insight into its role in osteogenesis.

## 5. Conclusion

OPN-coated surfaces promoted murine preosteoblast cell proliferation. In addition, cell spreading was markedly noted on the OPN-coated surfaces at early time points. Cell attachment was slightly altered. Thus, our results indicate that OPN coating influences cell proliferation and spreading. This may be useful for the modification of titanium implants to promote osteoblast response.

## Data Availability

The datasets used and/or analysed during the current study are available from the corresponding author upon reasonable request.
